# LncRNA EBLN3P Facilitates Osteosarcoma Metastasis by Enhancing Annexin A3 mRNA Stability and Recruiting HuR

**DOI:** 10.1245/s10434-023-14032-y

**Published:** 2023-08-19

**Authors:** Shengtao Wang, Xinxin Zeng, Peng Gui, Shujuan Xu, Zhaoxu Li, Dongxu Chen

**Affiliations:** 1 Department of Joint Surgery and Sports Medicine, Nanxishan Hospital of Guangxi Zhuang Autonomous Region, Xiangshan District, Guilin, Guangxi China; 2 Department of Pain, Banan Hospital of Chongqing Medical University, Banan District, Chongqing City, China; 3 Department of Trauma orthopedics and hand surgery, Nanxishan Hospital of Guangxi Zhuang Autonomous Region, Xiangshan District, Guilin, Guangxi China; 4 Department of Hematopathology, Affiliated Hospital of Guilin Medical University, Xiufeng District, Guilin, Guangxi China

**Keywords:** LncRNA EBLN3P, HuR, Annexin A3, Osteosarcoma, Proliferation, Migration, Invasion, Metastasis

## Abstract

**Background:**

Osteosarcoma (OS) represents a common type of bone cancer. Long non-coding RNAs (LncRNAs) have shown their potential in therapeutic modalities for OS. This study’s purpose was to reveal the action of lncRNA EBLN3P on OS growth and metastasis and its mechanism.

**Methods:**

Expressions of EBLN3P/Hu antigen R (HuR)/Annexin A3 (ANXA3) were determined by RT-qPCR/Western blot. Proliferation/migration/invasion of OS cells were assessed via CCK-8/Transwell assays after interfering EBLN3P/ANXA3/HuR. The co-localization of EBLN3P/ANXA3/HuR cells was observed by FISH/immunofluorescence assays. Interplays among EBLN3P/ANXA3/HuR and the half-life period of ANXA3 were assessed by RNA immunoprecipitation/RNA pull-down/RNA stability experiment. The nude mouse xenograft model was established, followed by EBLN3P treatment to assess the function of EBLN3P on OS.

**Results:**

EBLN3P/ANXA3 was highly expressed in OS cells. Silencing EBLN3P or ANXA3 limited the proliferation/migration/invasion of OS cells. Mechanically, EBLN3P/ANXA3 can bind to HuR, and EBLN3P enhanced ANXA3 mRNA stability by recruiting HuR, thus facilitating OS cell growth. Upregulated HuR or ANXA3 counteracted the suppressive action of silencing EBLN3P on OS cells. In vivo experiments revealed facilitated tumor growth and metastasis in vivo fomented by EBLN3P through manipulation of HuR/ANXA3.

**Conclusions:**

EBLN3P enhanced proliferative/migrative/invasive potentials of OS cells via increasing ANXA3 mRNA stability and protein level by recruiting HuR, which provided new potential therapeutic targets for OS clinical treatment. EBLN3P and ANXA3 might have potential roles in OS diagnosis, treatment, and prognosis. This study provided a theoretical reference for further clinical research in tumor surgery.

**Supplementary Information:**

The online version contains supplementary material available at 10.1245/s10434-023-14032-y.

Osteosarcoma (OS) represents the most widespread type of malignant tumor among adolescents, with three cases per million population and higher prevalence in males than females.^1^ The bone marrow end of the long bone, especially the distal femur and proximal humeral knee joint, is vulnerable to OS.^2^ OS can progress speedily and results in incredibly high mortality.^3,4^ Current therapies for OS consist largely of surgical operation, chemotherapy, and immunotherapy.^5^ Unfortunately, 30–50% of patients still face the risk of distant metastases.^6^ The strong metastatic ability of OS acts as a powerful factor in patient prognosis.^7^ Owing to the presented threats, understanding the OS metastasis mechanism will likely open new therapeutic options for OS.

Long non-coding RNAs (LncRNAs) with more than 200 bases in length have been widely acknowledged as regulators for tumor initiation and development.^8^ In specific, endogenous bornavirus-like nucleoprotein (EBLN3P), a newly discovered lncRNA located on chromosome 9: 37,079,935–37,086,874 forward strand,^9^ is highly-expressed in several cancers, such as OS,^10^ liver cancer,^11^ and colorectal cancer,^12^ and alteration of its expression may pose influences on tumor progression. Thus, it may be inferred that lncRNA EBLN3P plays a predominant role in OS development. The interaction between lncRNAs and specific RNA-binding protein (RBP) can enhance the stability of mRNA in the cytoplasm.^13,14^ One of the RBPs, Hu antigen R (HuR), is engaged in tumor progression in various ways. Representative examples are as follows. HuR enhances pancreatic cancer cell resistance to TRAIL by limiting the expression of death receptor 4.^15^ LINC00668 contributes to gastric cancer metastasis via combination with HuR-dependent PKN2 elevation.^16^ In addition, lncRNA B4GALT1-AS recruits HuR to promote YAP activity, thus fostering OS cell stemness and migration.^17^ However, the association between lncRNA EBLN3P and HuR in OS remains elusive.

Annexin A3 (ANXA3) belongs to the Annexin superfamily^18^ and participates in membrane transport, ion transport, cytoskeletal interaction, cell–cell signaling, inflammatory response, endothelial cell migration, adipocyte differentiation, and angiogenesis, and is closely connected to tumorigenesis.^19^ Past research has confirmed the abundant expression of ANXA3 in prostate cancer, lung cancer, and liver cancer,^20–22^ offering theoretical guides for tumor diagnosis and treatment. High expression of ANXA3 in OS cells has also been discovered in the preliminary study, eliciting OS cell apoptosis promotion after ANXA3 knockdown.^6^ Nevertheless, the mechanism of ANXA3 modulating OS is still pending. The puzzle of whether lncRNA EBLN3P affects OS metastasis via enhancement of ANXA3 mRNA stability and protein level by recruiting HuR remains unsolved even to this day. The purpose of the current study lies in investigating the molecular mechanism of EBLN3P in OS metastasis with the involvement of ANXA3 and HuR, in the hopes of providing a novel target for OS diagnosis and treatment.

## Patients and Methods

### Ethics Statement

All animal-associated experimental procedures were ratified by the Animal Ethics Committee of Nanxishan Hospital of Guangxi Zhuang Autonomous Region closely following the 8th Edition of the Guide for the Care and Use of Laboratory Animals published by the National Institutes of Health Publication in 2011. Confirming the study is reported in accordance with ARRIVE guidelines.

### Cell Culture and Grouping

Cells used in this study included human osteoblasts (hFOB1.19) and OS cell lines (HOS, U2OS, Saos2, and 143B), all of which were supplied by ATCC (Manassas, VA, USA) and were grown in Dulbecco’s modified Eagle’s medium consisting of 1% penicillin-streptomycin and 10% fetal bovine serum (FBS) (Gibco, Grand Island, NY, USA) in an incubator under an atmosphere of 5% CO_2_ at 37 C, and HOS and U2OS cells were allocated into the following groups: the si-NC (negative control) group, si-EBLN3P group, si-ANXA3 group, si-HuR group, pc-NC (pcDNA3.1 empty vector) group, pc-ANXA3 group, pc-HuR group, si-HuR + si-NC group, si-HuR + si-EBLN3P group, si-EBLN3P + pc-HuR group, and EBLN3P + pc-ANXA3 group. si-NC, si-EBLN3P, si-ANXA3, si-HuR, pc-NC, pc-ANXA3, and pc-HuR plasmids were designed and completed by GenePharma (Shanghai, China). The target sequences of siRNAs were as follows: si-NC: 5′-UUCUCCGAACGUGUCACGUTT-3′; si-EBLN3P: 5′-TGCAGGGCCAGTGATTGGTTT-3′; si-HuR, 5′-TTGTTAGTGTACAACTCATTT-3′; si-ANXA3, 5′-GATATCTCTCAAGCCTATTAT-3′. Lipofectamine 3000 (Invitrogen, Carlsbad, CA, USA) was deployed for cell transfection. The concentration of plasmids used for transfection was 800 ng/μL. Further experimentation was carried out after 48 h of cell transfection.

### RT-qPCR

TRIzol kits (Invitrogen) were employed for extraction of total RNA from aforementioned cells and tumor tissues. Reverse transcription was subsequently conducted using PrimeScript RT kits (TaKaRa, Dalian, China), followed by reverse transcription-quantitative polymerase chain reaction (RT-qPCR) using SYBR Premix Ex Taq II (TaKaRa) and 7500 Real-Time PCR system (Applied Biosystems, Foster City, CA, USA). The expression levels of EBLN3P, HuR, and ANXA3 were normalized to β-actin. The 2^−ΔΔCT^ method was adopted to obtain calculations. Three replicates were performed in each experiment. Primer sequences are as follows: EBLN3P, 5′-CAGACTAAAGGATCAAGCGAGA-3′ (forward) and 5′-ATCAATTGCCACAGGTTGAAGA-3′ (reverse); HuR, 5′-CCCTCTGGATGGTGGTGAAC-3′ (forward) and 5′-AAGCGGTTGAGAAAACGCAC-3′ (reverse); ANXA3, 5′-CCCATCAGTGGATGCTGAAG-3′ (forward) and 5′-TCACTAGGGCCACCATGAGA-3′ (reverse); β-actin, 5′-CATCCGTAAAGACCTCTATGCCAAC-3′ (forward) and 5′-ATGGAGCCACCGATCCACA-3′ (reverse).

### CCK-8 Assay

After seeding cells in 96-well plates (2 × 10^4^ cells/well) for 0 h, 24 h, 48 h, and 72 h, the cell counting kit-8 (CCK-8) assay kit (Beyotime, Shanghai, China) was utilized to assess the proliferative conditions. Elx800 Reader (Bio-Tek Instruments Inc., Winooski, VT, USA) was adopted for detection of absorbance at 450 nm.

### Transwell Invasion and Migration Assays

Transwell chamber was employed for the detection of OS cell invasion and migration. The invasive and migrative abilities were assessed utilizing Transwell chamber pre-filled with Matrigel matrix (BD Bioscience, Franklin Lakes, NJ, USA) and Matrigel-free chamber, respectively. Cells were starved for 24 h in a serum-free medium and subsequently resuspended in the medium with 1% FBS before experimentation. After seeding cells (1 × 10^5^) in the apical chamber, the basolateral chamber was added with 600 μL complete culture medium that contained 10% FBS. Following 24-h incubation, cells remaining in the apical chamber were wiped off with a cotton swab and fixed with 4% paraformaldehyde, followed by hematoxylin staining. Cells in five randomly selected visual fields were observed and counted.

### Western Blot

Radio immunoprecipitation assay lysis buffer that contained protease inhibitor was used for total protein extraction from cells and tissues, followed by protein concentration measurement utilizing bicinchoninic acid kits (Beyotime). After separation by sodium dodecyl sulfate-polyacrylamide gel electrophoresis (10%), 30 μg protein samples were transferred to polyvinylidene fluoride membranes (Millipore, Bedford, MA, USA). After blockage with 5% non-fat milk for 2 h, the membranes were incubated with primary antibodies ANXA3 (1/1000, ab228761, Abcam), HuR (1:5000, ab200342), and β-actin (1/5000, ab6276, Abcam) for one night at 4 °C. Following membrane washing with Tris-buffered saline-Tween-20, HRP-labeled goat anti-rabbit secondary antibody immunoglobulin G (IgG) (1/2000, ab205718, Abcam) was added to incubate with membranes for 1 h at room temperature, followed by color development using enhanced chemiluminescence. Gray value of each protein band was analyzed utilizing Image J (Media Cybernetics, Rockville, MD, USA). β-actin acted as an internal reference. Three replicates were performed on each experiment.

### Fluorescent In Situ Hybridization (FISH) and Immunofluorescence Double Staining Assays

FISH assay was conducted using Cyanine3-labelled nucleic acid probes with specific complementary lncRNA EBLN3P or ANXA3 mRNA (Ribobio, Guangzhou, Guangdong, China). Cell climbing slides of HOS and U2OS were prepared, fixed with 4% paraformaldehyde at 4 °C for 10 min, washed with phosphate-buffered saline (PBS), added with 0.1% Triton-X-100 to break the membranes, and placed at room temperature for 15 min. The samples were then added with 5 μL of lncRNA EBLN3P or ANXA3 mRNA nucleic acid probe hybridization solution and incubated overnight at room temperature in the dark. After a rinse with PBS, the samples were blocked with 3% bovine serum albumin, incubated overnight with HuR primary antibody (1:500, ab200342) at 4 °C in the dark, and incubated at room temperature using Alexa Fluor®488-coupled IgG secondary antibody (1:1000, ab150077) in the dark for 1 h. Thereafter, cell nuclei were re-stained with 4′,6-diamidino-2-phenylindole (Sigma-Aldrich, St. Louis, MO, USA) and incubated at room temperature in the dark for 5 min. After washing with PBS, the sections were sealed with glycerol, and the co-localization of lncRNA EBLN3P or ANXA3 mRNA with HuR was observed using an inverted fluorescence microscope (Hitachi Limited, Tokvo, Japan).

### RNA Immunoprecipitation (RIP) Assay

OS cells were lysed using 25 mmol/L Tris-HC1 buffer (pH 7.5) and added with 100 U/mL RNase inhibitor (Sigma-Aldrich), and then incubated at 4 °C with protein A Sepharose beads pre-coated with 3 μg HuR antibody or its control IgG for 1.5 h. RNA-protein complex was pulled down by protein A/G agarose beads, followed by RNA extraction using TRIzol reagent. Ultimately, expression levels of EBLN3P or ANXA3 were determined by RT-qPCR.

### RNA Pull-Down

T7 RNA polymerase (Ambio Life, Shanghai, China) was adopted for transcription of EBLN3P sense or antisense and ANXA3 sense or antisense, followed by purification using RNeasy Plus Mini Kit (Qiagen, Hilden, Germany). EBLN3P or ANXA3 was treated with RNase-free Dnase I (Qiagen). Afterwards, EBLN3P sense or antisense and ANXA3 sense or antisense were biotin-labeled with biotin RNA labeling mixture (Ambio Life). RNA pull-down assay was carried out later using Pierce^TM^ Magnetic RNA-Protein Pull-Down kit (Thermo Scientific Pierce, Waltham, MA, USA) as per the provided protocols. Finally, EBLN3P enrichment was estimated by Western blot.

### RNA Stability Assay

After introducing si-EBLN3P, si-HuR, or their negative controls into OS cells, OS cells were treated with 1 μg/mL actinomycin D. Cells were harvested after 1 h, 2 h, 4 h, and 6 h, followed by detection of ANXA3 mRNA expression by means of RT-qPCR.

### Nude Mouse Model of Xenograft Tumor

The 4-week-old female Balb/c nude mice from Vital River Laboratory Animal Technology [Beijing, China; SCXK(Beijing) 2016-0006] were acclimated for 1 week under controlled conditions (24–26 °C, 50–60% humidity, ad libitum access to water and feed) prior to experimentation.

Stably transfected U2OS cells were screened as follows. First, U2OS cells were infected by lentivirus loaded with si-EBLN3P plasmid or simultaneously infected with pc-ANXA3 (containing puromycin-resistant gene). Then, U2OS cells were selected with 5 μg/mL puromycin for 48 h and added to a medium supplemented with 1 μg/mL puromycin to expand cells. Cells were frozen until use after RT-qPCR confirmed the stable low expression of EBLN3P or overexpression of ANXA3. Mice were randomized to the si-NC group and si-EBLN3P group, and the si-EBLN3P + pc-NC group and si-EBLN3P + pc-ANXA3 group, with six mice per group. U2OS cells (1 × 10^5^ cells/mL, 100 μL) stably transfected with si-NC, si-EBLN3P, si-EBLN3P + pc-NC, and si-EBLN3P + pc-ANXA3 lentiviruses were injected subcutaneously into each mouse. The tumor volume was measured on days 7, 14, 21, and 28, respectively, using the equation: [V = 0.5 × L (length) × W (width)^2^], and 4 weeks later, excessive pentobarbital sodium (800 mg/kg) was intraperitoneally injected into each nude mouse for euthanasia. Consequently, tumors were removed and weighed and analyzed further.

### Tumor Metastasis In Vivo

For assessment of tumor metastasis in vivo, U2OS cells (1×10^6^ cells/mL, 100 μL) stably transfected with si-NC, si-EBLN3P, si-EBLN3P + pc-NC, and si-EBLN3P + pc-ANXA3 lentiviruses were injected into each mouse via the caudal vein, and 4 weeks later, nude mice were euthanized, with lung tissues isolated for photographing. The metastatic nodes in lung tissues of mice were counted.

### Immunohistochemistry

Tumor tissues were sectioned and incubated with Ki-67 antibody (1/1000, ab279653, Abcam), followed by staining with horseradish peroxidase anti-rabbit IgG and diaminobenzidine. Subsequently, after incubation with mouse secondary antibody, EnVision G2 System/AP Rabbit Mouse Permanent Red (Dako, Glostrup, Denmark) was utilized to stain the sections, followed by counterstaining using hematoxylin. A fluorescent inverted microscope (Hitachi Limited, Tokyo, Japan) was utilized for observation.

### Statistical Analysis

The obtained values were depicted as mean ± standard deviation (SD). GraphPad Prism 8 software was deployed for data analyses and plotting. The independent sample *t*-test was utilized for data comparison between two groups, and one-way analysis of variance (ANOVA) together with Tukey’s multiple comparisons test was utilized for multigroup data comparison. At least three replicates were guaranteed in each cell experiment. The difference of *p* < 0.05 represented statistical significance.

## Results

### Silencing EBLN3P Impeded OS Cell Multiplication, Migration, and Invasion

**Fig. 1:**
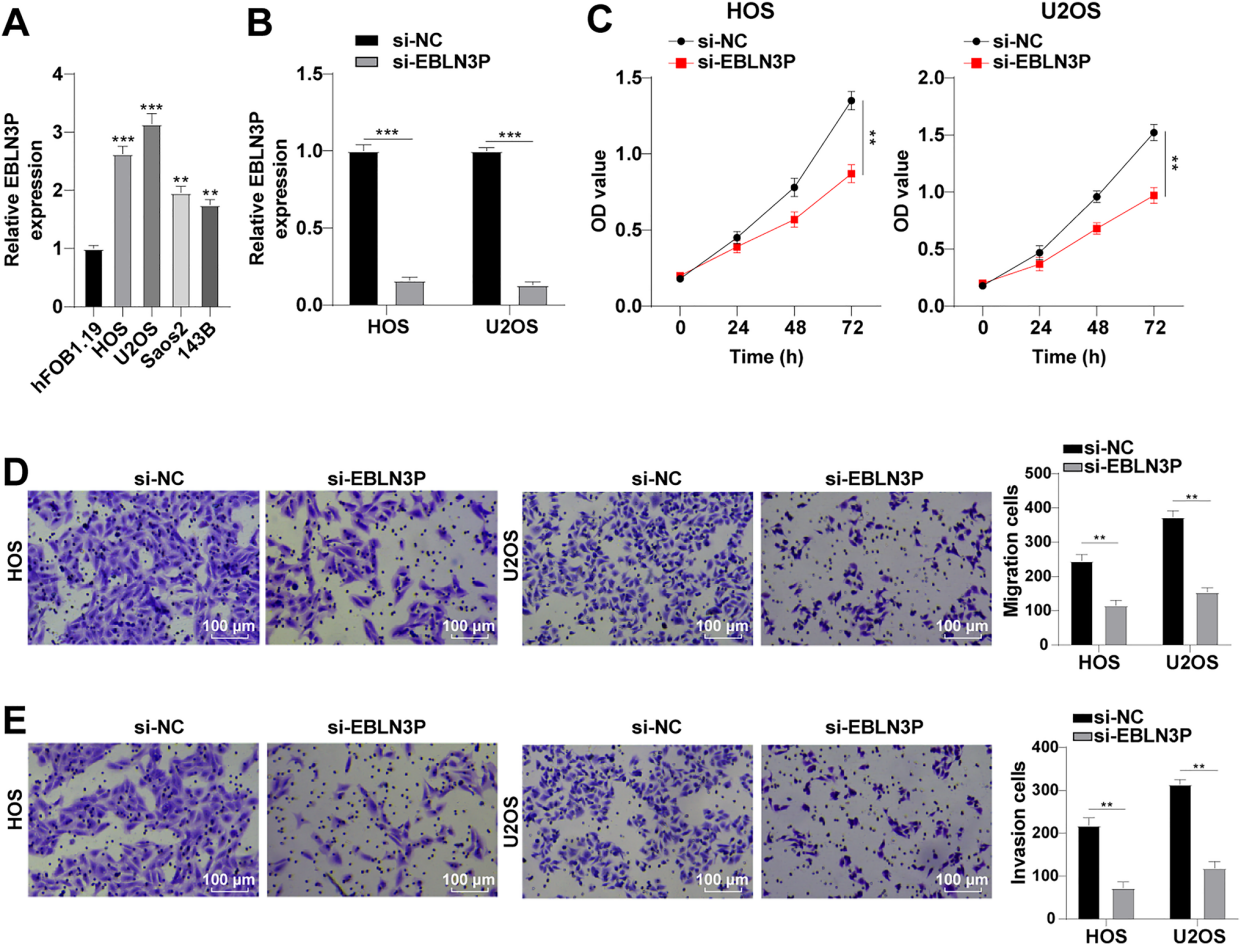
Silencing EBLN3P impeded OS cell proliferation, migration, and invasion; **A** EBLN3P expression in hFOB1.19, HOS, U2OS, Saos2, and 143B cells determined via RT-qPCR; **B** EBLN3P expression determined via RT-qPCR; **C** proliferative ability of OS cells assessed via CCK-8; **D** migrative ability of OS cells assessed via Transwell assay; and **E** invasive ability of OS cells assessed via Transwell assay; three replicates were guaranteed in each cell experiment, with data presented as mean ± SD; the independent sample *t*-test was utilized to compare data between two groups, and one-way ANOVA was applied to compare data among multiple groups with Tukey’s test as post hoc test; **p < 0.01, ***p < 0.001

As aforementioned, EBLN3P is dysregulated in OS.^10^ We therefore further clarified this finding by detecting EBLN3P expression in osteoblasts hFOB1.19 and OS cells via RT-qPCR, which showed an increase in EBLN3P expression in OS cells, especially HOS and U2OS cells (Fig. 1A). For this reason, we selected HOS and U2OS cells for further experimentation. With the intention of evaluating the action of EBLN3P on OS cells, si-EBLN3P was introduced in HOS and U2OS cells to silence EBLN3P (Fig. 1B). Subsequently, CCK-8 confirmed the suppressive function of silencing EBLN3P on OS cell proliferation (Fig. 1C). The same inhibitory role of EBLN3P was found on OS cell migration (Fig. 1D) and invasion (Fig. 1E) on the basis of the results of Transwell assays. Jointly, silencing EBLN3P could suppress proliferation, migration, and invasion of OS cells.

### ANXA3 Knockdown Repressed OS Cell Growth

**Fig. 2:**
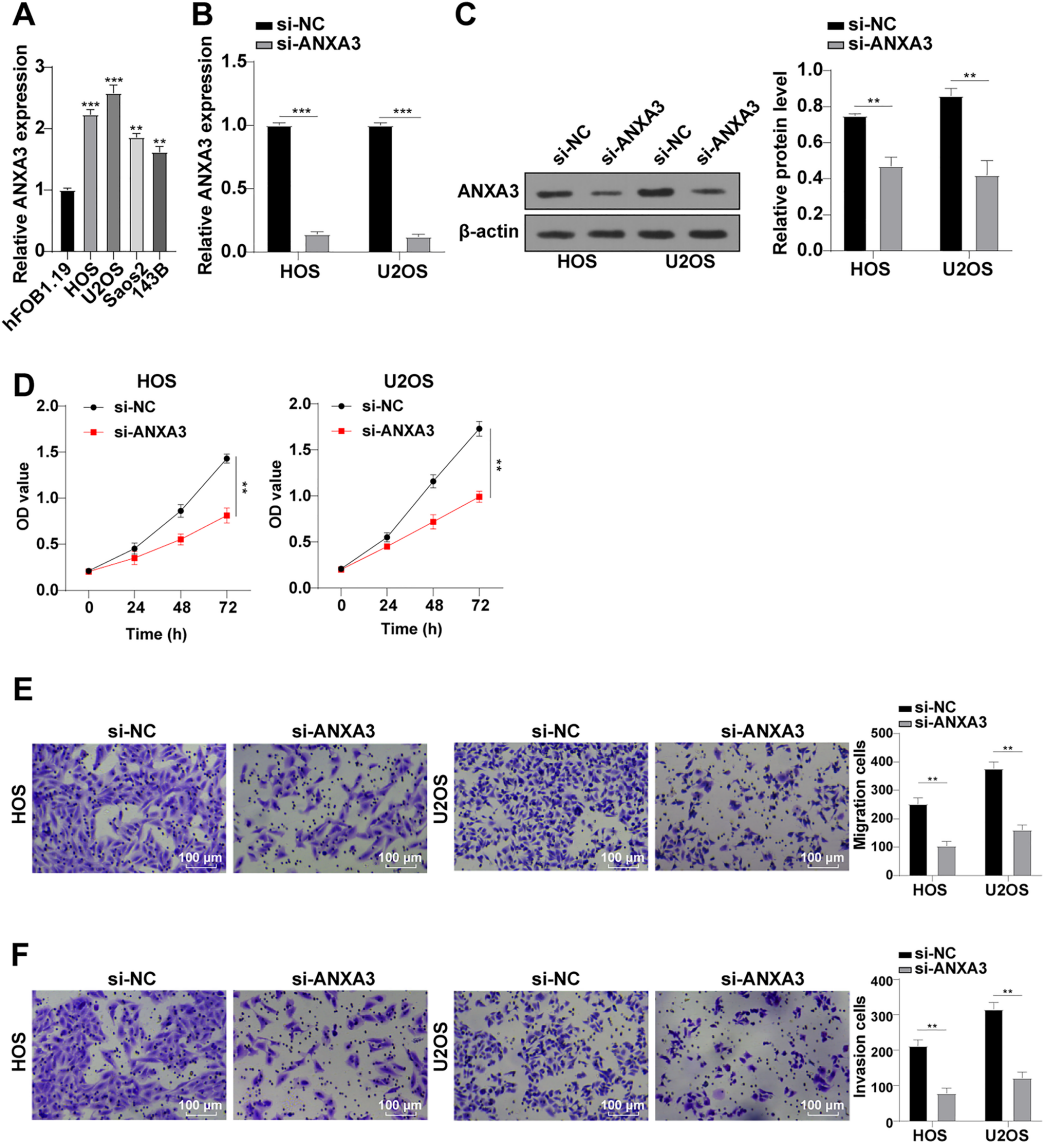
ANXA3 knockdown repressed OS cell proliferation, migration, and invasion; **A** ANXA3 expression in hFOB1.19, HOS, U2OS, Saos2, and 143B cells determined via RT-qPCR; **B** ANXA3 expression determined via RT-qPCR; **C** ANXA3 expression determined via Western blot; **D** proliferative ability of OS cells assessed via CCK-8; **E** migrative ability of OS cells assessed via Transwell assay; and **F** invasive ability of OS cells assessed via Transwell assay; three replicates were guaranteed in each cell experiment, with data presented as mean ± SD; the independent sample *t*-test was utilized to compare data between two groups, and one-way ANOVA was applied to compare data among multiple groups with Tukey’s test as post hoc test; **p < 0.01, ***p < 0.001

ANXA3 is aberrantly expressed in various kinds of tumors and shows an association with cancer progression.^23^ The elevated ANXA3 level in osteosarcoma and its correlation with cell apoptosis have been reported.^6^ To investigate the function of ANXA3 in OS, we herein measured ANXA3 expression in OS cells by means of RT-qPCR and found an increasing trend of ANXA3 expression (Fig. 2A). Afterwards, ANXA3 was knocked down by siRNA to observe what changes were brought about to OS cell proliferation, migration, and invasion. Following si-ANXA3 transfection, HOS and U2OS cells exhibited decreased ANXA3 expression (Fig. 2B, C). In comparison with the si-NC group, the si-ANXA3 group presented considerably inhibited proliferation (Fig. 2D), migration (Fig. 2E), and invasion (Fig. 2F) in HOS and U2OS cells. All in all, ANXA3 knockdown substantially suppressed OS cell proliferation, migration, and invasion.

### EBLN3P and ANXA3 Bound to HuR

**Fig. 3:**
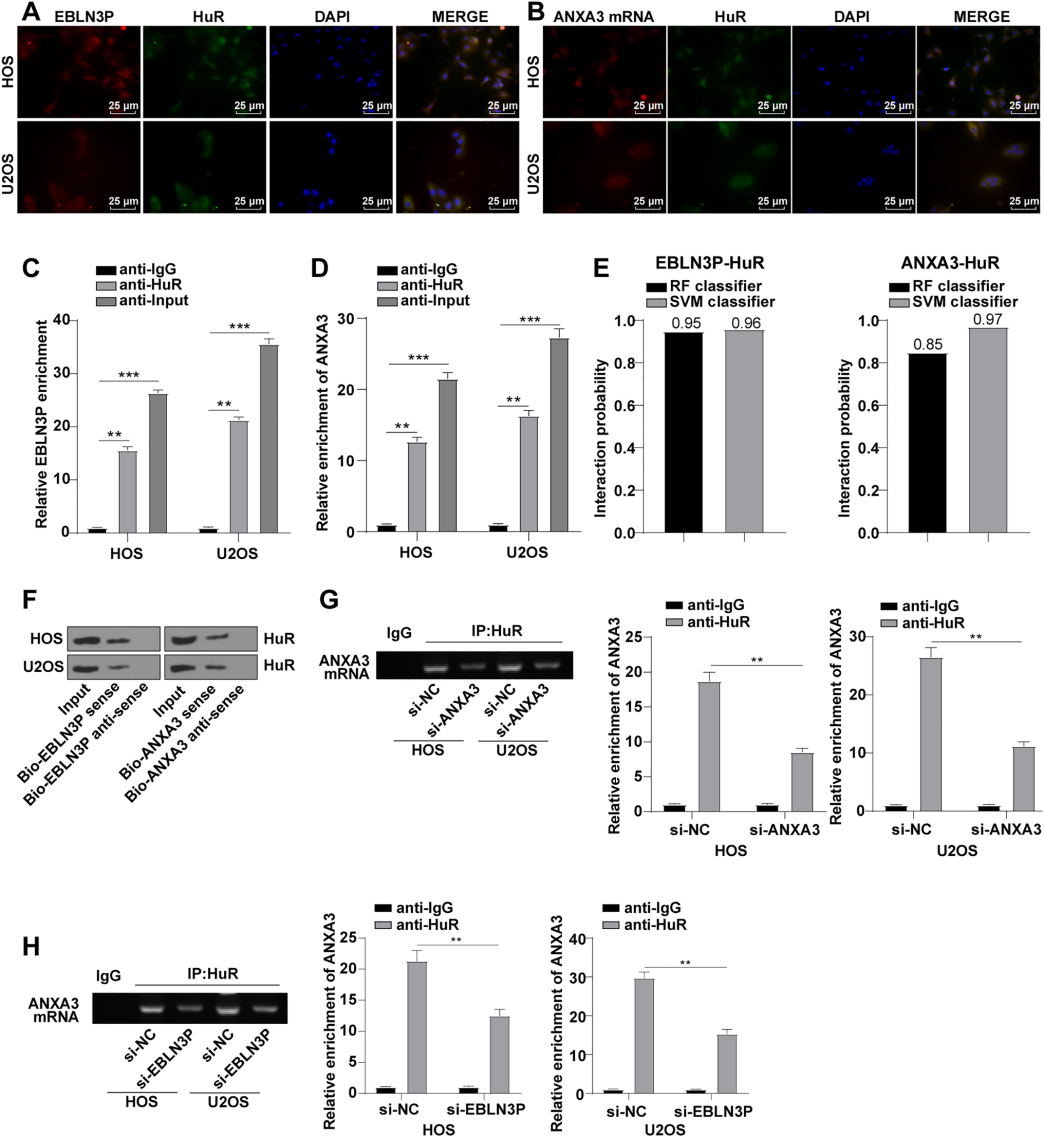
EBLN3P and ANXA3 bound to HuR; **A**/**B** cellular co-localization of EBLN3P or ANXA3 with HuR protein in OS cells was observed by FISH and immunofluorescence staining; **C**, **D** relative EBLN3P or ANXA3 enrichment in HuR-, IgG, or Input-coupled magnetic beads in OS cells detected via RIP assay; **E** RF and SVM classifier scores predicted on RPISeq website, with scores > 0.5 indicating higher binding ability; **F** binding of EBLN3P (or ANXA3) to HuR in OS cells assessed via RNA pull-down; and **G**, **H** relative ANXA3 enrichment in OS cells with HuR- or IgG-coupled magnetic beads after ANXA3 knockdown (or silencing EBLN3P) detected via RIP assay; three replicates were guaranteed in each cell experiment, with data presented as mean ± SD; the independent sample *t*-test was utilized to compare data between two groups, and one-way ANOVA was applied to compare data among multiple groups with Tukey’s test as post hoc test; **p < 0.01, ***p < 0.001

On the basis of previous conclusions, we next sought to know the regulatory relationship between EBLN3P and ANXA3 in OS. HuR, as an RBP, is engaged in the development of diverse cancers, including OS.^17,24^ Thereby, we conducted FISH and immunofluorescence assays and observed that EBLN3P and HuR proteins were co-localized in the cytoplasm (Fig. 3A), and ANXA3 mRNA and HuR were also co-localized in the cytoplasm (Fig. 3B). Thereafter, we performed the RIP assay to detect HuR enrichment in EBLN3P and ANXA3 mRNA, which showed evident enrichment of EBLN3P in HuR-labeled beads in HOS and U2OS cells relative to normal IgG (Fig. 3C), and significant enrichment of ANXA3 in HuR-labeled beads (Fig. 3D), indicative of an interaction between EBLN3P or ANXA3 and HuR protein. Moreover, the binding ability between EBLN3P and HuR or ANXA3 and HuR was further predicted using RNA-Protein Interaction Prediction (RPISeq) (http://pridb.gdcb.iastate.edu/RPISeq/). RPISeq consists of two variants: RPISeq-RF (using random forest classifier) and RPISeq-SVM (using support vector machine classifier). Scores of both RF and SVM classifiers > 0.5 are indicative of the high binding ability of the molecule. RPISeq website showed the scores of RF and SVM classifiers of EBLN3P and HuR at 0.95 and 0.96, respectively, and those of ANXA3 and HuR at 0.85 and 0.97, respectively (Fig. 3E), which signified that EBLN3P (or ANXA3) and HuR had high potential binding ability. RNA pull-down suggested the presence of HuR enrichment in the Bio-EBLN3P sense group (or Bio-ANXA3 sense group), which further validated the binding of EBLN3P (or ANXA3) to HuR protein (Fig. 3F). In addition, RIP assay revealed a decrease in HuR enrichment in ANXA3 mRNA after ANXA3 knockdown or silencing EBLN3P in OS cells (Fig. 3G, H), which verified the interplay between ANXA3 and HuR. Taken conjointly, both EBLN3P and ANXA3 could bind to HuR.

### EBLN3P Stabilized ANXA3 by Binding to HuR

**Fig. 4:**
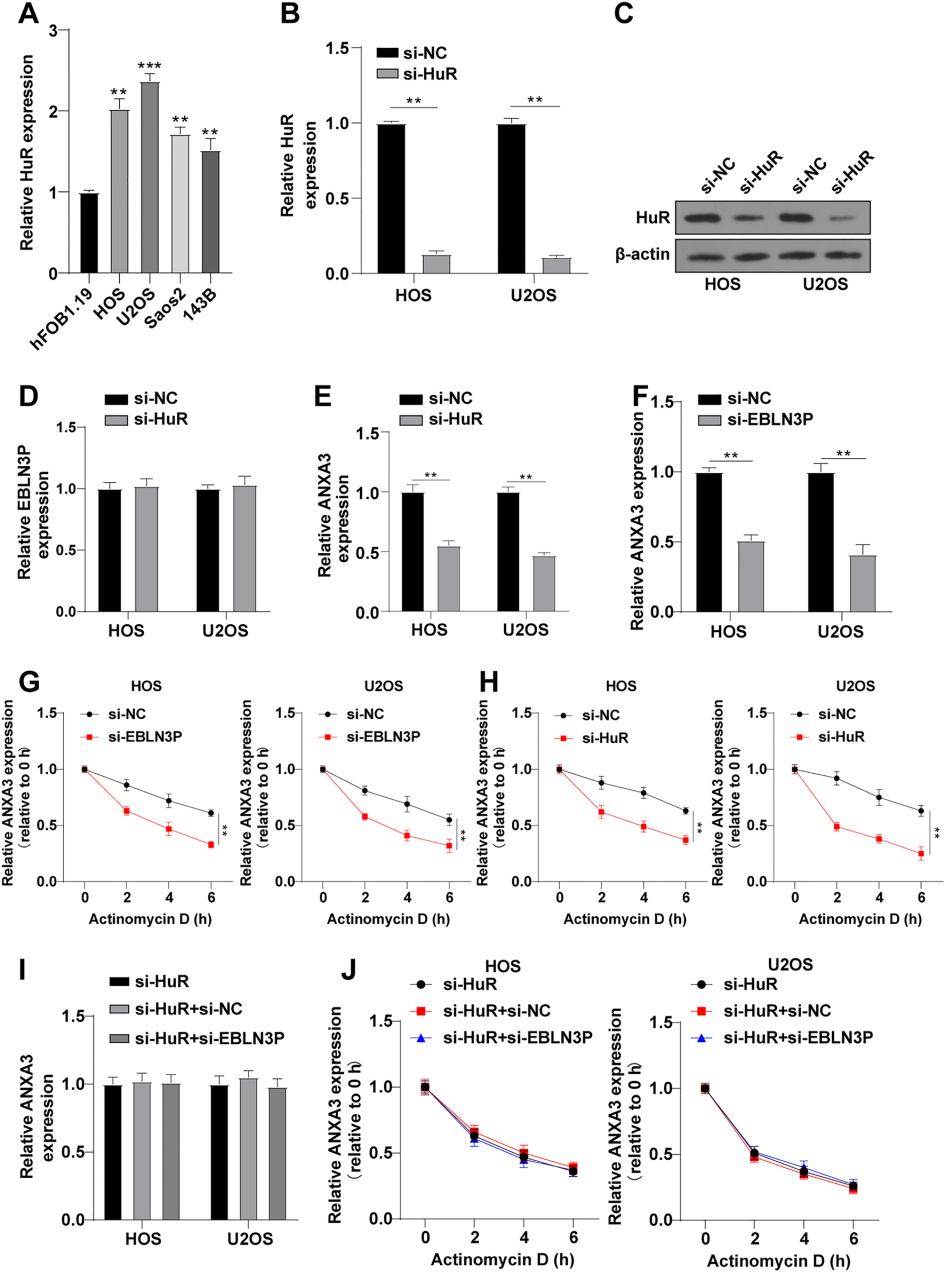
EBLN3P stabilized ANXA3 by binding to HuR; **A** HuR expression in hFOB1.19, HOS, U2OS, Saos2, and 143B cells determined via RT-qPCR; **B**, **C** HuR expression determined via RT-qPCR (**B**) and Western blot (**C**); **D** EBLN3P expression determined via RT-qPCR; **E**, **F** ANXA3 mRNA expression determined via RT-qPCR; **G**, **H** degradation rate of ANXA3 mRNA in OS cells after silencing EBLN3P or HuR depletion detected via RNA stability assay; **I** ANXA3 mRNA expression determined via RT-qPCR; and **J** degradation rate of ANXA3 mRNA via RNA stability assay; three replicates were guaranteed in each cell experiment, with data presented as mean ± SD; the independent sample *t*-test was utilized to compare data between two groups, and one-way ANOVA was applied to compare data among multiple groups with Tukey’s test as post hoc test; **p < 0.01, ***p < 0.001

Recent evidence suggests that lncRNAs can modulate mRNA expression by recruiting RBPs.^25,26^ On the basis of the above results and the regulatory mechanism of RBPs, we speculated that EBLN3P might regulate ANXA3 expression by binding to HuR. To validate this hypothesis, we first determined HuR expression in osteoblasts hFOB1.19 and OS cells, and found highly expressed HuR in HOS and U2OS cells (Fig. 4A). After depleting HuR via si-HuR transfection, the mRNA and protein level of HuR was considerably decreased (Fig. 4B, C). Intriguingly, EBLN3P expression remain unchanged after si-HuR transfection (Fig. 4D), while the ANXA3 mRNA levels were diminished (Fig. 4E), suggesting that EBLN3P might be located in the upstream of the EBLN3P-HuR-ANXA3 axis. Additionally, the function of EBLN3P on expression levels of ANXA3 was assessed. As presented in Fig. 4F, ANXA3 levels declined after silencing EBLN3P. To further ascertain the EBLN3P-HuR-ANXA3 axis, RNA stability assay was carried out, which demonstrated that the half-life period of ANXA3 mRNA in OS cells was reduced after silencing EBLN3P or HuR depletion (Fig. 4G, H). In addition, ANXA3 expression level remained unchanged in OS cells stably transfected with si-HuR after silencing EBLN3P (Fig. 4I), and so did the degradation rate of ANXA3 mRNA (Fig. 4J). These results illustrated that EBLN3P modulated ANXA3 in a HuR-dependent manner. Jointly, EBLN3P could stabilize ANXA3 mRNA via binding to HuR.

### EBLN3P Facilitated OS Cell Growth by Upregulating ANXA3 in a HuR-Dependent Manner

**Fig. 5:**
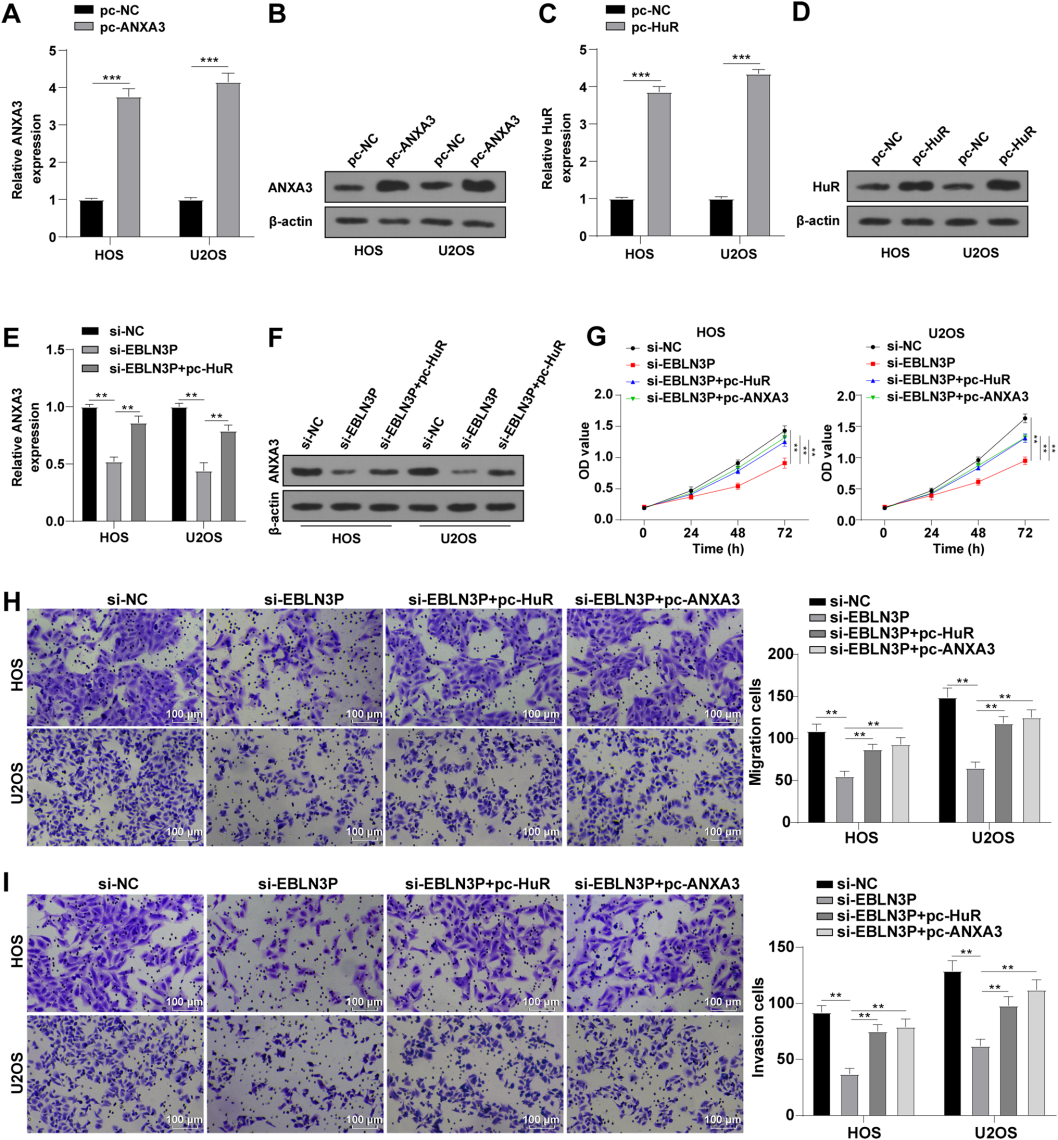
EBLN3P facilitated OS cell growth by upregulating ANXA3 in a HuR-dependent manner; **A**, **B** ANXA3 expression determined via RT-qPCR (**A**) and Western blot (**B**); **C**, **D** HuR expression determined via RT-qPCR (**C**) and Western blot (**D**); **E**, **F** ANXA3 expression determined via RT-qPCR (**E**) and Western blot (**F**); **G** proliferative ability of OS cells assessed via CCK-8; **H** migrative ability of OS cells assessed via Transwell assay; and **I** invasive ability of OS cells assessed via Transwell assay; three replicates were guaranteed in each cell experiment, with data presented as mean ± SD; the one-way ANOVA was applied to compare data among multiple groups with Tukey’s test as post hoc test; **p < 0.01, ***p < 0.001

We subsequently conducted a combined experiment to further verify the obtained results. Firstly, RT-qPCR and Western blot were conducted to confirm the efficiency of pc-ANXA3 and pc-HuR transfection, which demonstrated elevated mRNA and protein levels of ANXA3 and HuR following transfection (Fig. 5A–D), suggestive of successful transfection. In addition, overexpressed HuR to some extent abrogated the downregulated ANXA3 levels induced by silencing EBLN3P (Fig. 5E, F). Moreover, overexpression of HuR or ANXA3 to a certain extent abrogated the inhibitory effect of silencing EBLN3P on proliferation (Fig. 5G), migration (Fig. 5H), and invasion (Fig. 5I) of OS cells. Therefore, it could be inferred that EBLN3P upregulated ANXA3 in a HuR-dependent manner and to some extent facilitated OS cell proliferation, migration, and invasion.

### EBLN3P Contributed to OS Growth and Metastasis

**Fig. 6:**
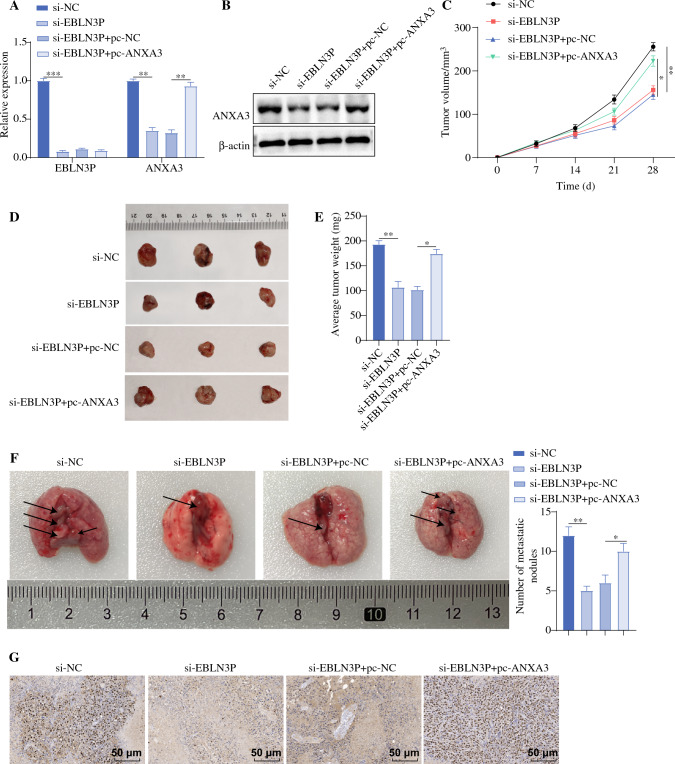
EBLN3P contributed to OS growth and metastasis; **A** EBLN3P and ANXA3 expression levels in tumor tissues of nude mice were assessed by RT-qPCR; **B** ANXA3 expression in tumor tissues of nude mice were determined by Western blot; **C** measurement of tumor volume; **D** picture of tumors isolated from nude mice; **E** measurement of tumor weight; **F** number of lung metastatic nodes; and **G** Ki-67-positive expression determined via immunohistochemistry, *N* = 6, *p < 0.05, **p < 0.01, ***p < 0.001

Finally, U2OS cells were injected into nude mice to ascertain the function of EBLN3P on the growth and metastasis of tumors. Relative to the si-NC group, the si-EBLN3P group had diminished expression levels of EBLN3P and ANXA3 in nude mouse tumor tissues (Fig. 6A, B), decelerated tumor growing speed, reduced tumor weight (Fig. 6C–E), and reduced metastatic nodes in lung tissues (Fig. 6F). Immunohistochemistry revealed noticeably decreased Ki-67-positive expression in the si-EBLN3P group compared with that in the si-NC group (Fig. 6G). However, overexpressing ANXA3 while silencing EBLN3P (Fig. 6A, B) increased the tumor growth rate and weight in nude mice (Fig. 6C–E), the number of pulmonary metastatic nodules (Fig. 6F), and Ki-67 positive expression levels (Fig. 6G). The in vivo assay further confirmed that silencing EBLN3P could downregulate ANXA3 and suppress OS growth and metastasis.

**Fig. 7:**
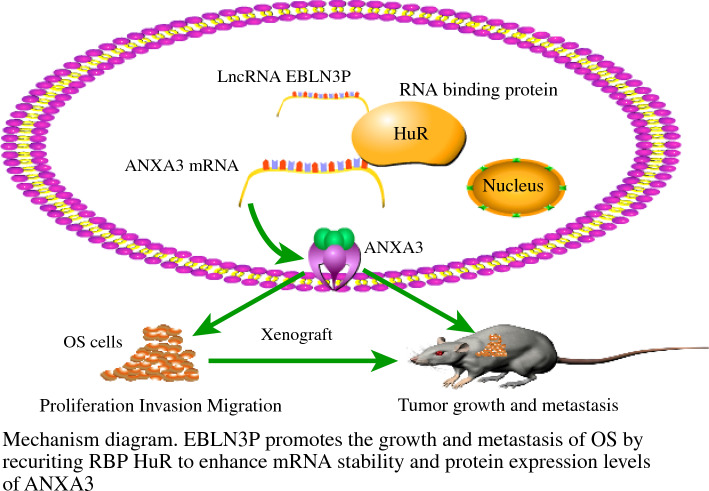
Mechanism diagram of EBLN3P facilitating OS metastasis by enhancing ANXA3 mRNA stability and protein level through HuR recruitment; *OS* osteosarcoma, *RBP* RNA-binding protein

Overall, EBLN3P enhanced ANXA3 mRNA stability and protein levels by recruiting RBP HuR, thereby promoting the growth and metastasis of OS (Fig. 7).

## Discussion

Metastasis is an enormous challenge in OS management and an indicator of poor prognosis.^27^ Several researchers have investigated the mechanism of lncRNA EBLN3P in metastasis of liver cancer,^11^ which triggered our interest in the mechanism of lncRNA EBLN3P in OS metastasis. This paper managed to preliminarily explain that lncRNA EBLN3P facilitated OS metastasis by recruiting HuR and enhancing ANXA3 mRNA stability and protein level (Fig. 7).

Increased expression of lncRNA EBLN3P has been documented in OS cell lines^10^ and exhibits a close link to the prognosis of patients with OS.^28^ To understand the role of EBLN3P in the cellular process of OS development, the first step was to examine the expression pattern of EBLN3P in OS cells. Obviously, EBLN3P was highly expressed in OS cells, specifically in HOS and U2OS cells, relative to that in osteoblasts hFOB1.19. For this reason, we selected HOS and U2OS cells as study subjects and silenced EBLN3P to observe the changes brought to those cells. Similarly, EBLN3P knockdown substantially impedes the proliferation and migration of colorectal carcinoma cells.^12^ Likewise, silencing EBLN3P exerted an inhibitory effect on the proliferative, migrative, and invasive capabilities of OS cells. Subsequently, we overexpressed EBLN3P in 143B OS cells with relative low EBLN3P expression and discovered salient increases in proliferative activity, migration, and invasion abilities (Supplementary Fig. 1), further confirming EBLN3P as a carcinogenic factor for OS.

The expression pattern of ANXA3 in OS cell line HOS has been documented earlier.^29^ We further confirmed the increased expression of ANXA3 in OS cell lines HOS, U2OS, Saos2, and 143B. In the same way, we depleted ANXA3 expression in OS cells to investigate its role in OS cell behaviors. Downregulation of ANXA3 plays an essential role in decreasing cell proliferation and invasion in breast cancer^30^ and gastric cancer.^31^ The same inhibitory action was produced by ANXA3 knockdown on OS cells.

Since we have found the common ground of EBLN3P and ANXA3 in OS cells, the next step was to investigate whether there is a regulatory relationship between EBLN3P and ANXA3 in OS. LncRNA TUG1 potentiates bladder cancer development and metastasis by activating Annexin A8.^32^ LncRNA XIST has been shown to bind to HuR and thus regulate AGO2 expression in OS.^33^ On the basis of the existing evidence, we assumed the engagement of HuR in the regulation of OS cells mediated by EBLN3P and ANXA3. The interaction between EBLN3P and ANXA3 and HuR was verified through the observed enrichment of EBLN3P and ANXA3 in HuR-labeled beads. Taken together, both EBLN3P and ANXA3 could bind to HuR. However, the association between EBLN3P and ANXA3 remains unclear. Several studies have reported that lncRNAs can modulate mRNA stability of mRNAs via HuR.^26,34,35^ We subsequently measured HuR expression in OS cells to know its role in their interaction. HuR expression was increased in HOS and U2OS cells. After HuR depletion, EBLN3P levels remained unchanged while ANXA3 mRNA and protein levels were downregulated. The same expression pattern of ANXA3 was observed after silencing EBLN3P. Knockdown of EBLN3P or HuR considerably reduced the half-period of ANXA3 mRNA in OS cells. EBLN3P knockdown in OS cells with HuR depletion caused no significant alterations in ANXA3 expression and the degradation rate of ANXA3 mRNA. Furthermore, EBLN3P can modulate the proliferation of lung adenocarcinoma cells through the miR-655-3p/Bcl2 axis.^36^ Knockdown of EBLN3P hinders the proliferation, invasion, and migration of Jurkat cells by increasing miR-655-3p expression.^37^ As for HuR, there is evidence that HuR-RNA interactions play essential roles in cancer treatment.^38^ Additionally, the interplay between HuR and microRNAs in gene regulation is implicated in angiogenesis.^39^ There is no report on the regulation of lncRNA EBLN3P on HuR. Herein, a likely mechanism that lncRNA EBLN3P could stabilize ANXA3 mRNA by binding to HuR was proposed in this study.

What follows is the action of HuR on OS cells. Researchers have noticed the contributory role of HuR in OS progression.^40^ We found that the declined ANXA3 expression induced by silencing EBLN3P was partially counteracted by HuR overexpression. Furthermore, the limitation on proliferative, migrative, and invasive abilities of OS cells fomented by silencing EBLN3P was partly nullified by overexpressed HuR or ANXA3. The deletion of HuR could jeopardize migration, invasion, and stemness of OS cells.^41^ Collectively, EBLN3P upregulated ANXA3 and thus accelerated OS cell growth via a HuR-dependent mechanism. The final step of this study was to validate the known facts via in vivo experiments. As expected, xenograft nude mice transfected with si-EBLN3P displayed reduced tumor growth and weight, metastatic nodes in lung tissues, and positive expression of Ki-67, along with downregulated EBLN3P and ANXA3. These results ascertained that silencing EBLN3P could suppress OS growth and metastasis by downregulating ANXA3, which resembled the past research on EBLN3P facilitating liver cancer development in vitro.^11^

Overall, lncRNA EBLN3P contributed to OS progression and metastasis by recruiting HuR to enhance the mRNA stability and protein level of ANXA3. This study provided new potential therapeutic targets for the clinical treatment of OS. EBLN3P and ANXA3 might have potential roles in the diagnosis, treatment, and prognosis of OS, and this study provided a theoretical reference for further clinical research in tumor surgery. Nonetheless, this study has several limitations. This study lacked clinical research data and we did not conduct complete mechanism validation experiments in OS cells with relatively low EBLN3P expression. Furthermore, despite the finding of the EBLN3P/HuR/ANXA3 axis in OS in providing epigenetic evidence for OS occurrence and development, the downstream pathways associated with the malignant behaviors of OS cells have not been explored yet, and clinical verification is lacking. Thereupon, the direction of future studies shall lie on the epigenetic mechanism by which EBLN3P affects OS progression via regulating the HuR/ANXA3 axis through downstream pathways.

### Supplementary Information

Below is the link to the electronic supplementary material.

Supplementary file1 (DOCX 20 KB)

Supplementary file2 (TIFF 6376 KB)

## Data Availability

The data that support the findings of this study are available from the corresponding author upon reasonable request.
