# Supplementary material for: LncRNA EBLN3P Facilitates Osteosarcoma Metastasis by Enhancing Annexin A3 mRNA Stability and Recruiting HuR
Source: Ann Surg Oncol. 2023 Aug 19;30(13):8690–703. doi: 10.1245/s10434-023-14032-y (PMC10625973; doi:10.1245/s10434-023-14032-y)
Supplement: Supplementary file 1 — Supplementary file1 (DOCX 20 KB) [file 10434_2023_14032_MOESM1_ESM.docx]

**Supplementary experiment**

**Experimental methods**

OS cell line 143B cells with relative low EBLN3P expression was seeded in 6-well plates (1 × 10^6^/well). Upon 80% confluence, cells were manipulated with overexpression plasmid of EBLN3P (oe-EBLN3P group) and empty plasmid (oe-NC group) using Lipofectamine 3000 following instructions, with the transfection plasmid concentration of 800 ng/μL. After 48-h transfection, the expression changes of EPLN3P were determined by RT-qPCR, cell proliferative activity was assessed by CCK-8, and migration and invasion abilities were evaluated by Transwell assay (the specific methods are shown in the Methods section of the manuscript).

**Results**

**EBLN3P overexpression promoted 143B cell invasion, migration and proliferation**

The overexpression plasmid of EBLN3P was introduced into OS cell line 143B with relative low expression of EBLN3P to up-regulate EBLN3P expression. As reflected by RT-qPCR results, there was an obvious increase in EBLN3P expression in the oe-EBLN3P group (Supplementary Figure 1A). CCK-8 elicited a marked increase in cell proliferative activity in the oe-EBLN3P group (Supplementary Figure 1B). Transwell assay revealed a salient increase in the number of cells that migrated and invaded in the oe-EBLN3P group. Overall, EBLN3P overexpression could promote the proliferation, migration, and invasion of OS cells, which further confirmed that EBLN3P was one of the carcinogenic factors for OS.

**Figure**

**Supplementary Figure 1** EBLN3P overexpression enhanced 143B cell proliferation, migration and invasion. A: The relative expression of EBLN3P in 143B cells was assessed by RT-qPCR; B: 143B cell proliferative activity changes were assessed by CCK-8; C: 143B cell migration and invasion abilities were evaluated by Transwell assay. Cell experiment was repeated thrice. Data were represented as mean ± standard deviation and tested by independent *t* test between two groups. ***p* < 0.01, ****p* < 0.001.
