# Supplementary figures and images for: LncRNA EBLN3P Facilitates Osteosarcoma Metastasis by Enhancing Annexin A3 mRNA Stability and Recruiting HuR
Source: Ann Surg Oncol. 2023 Aug 19;30(13):8690–703. doi: 10.1245/s10434-023-14032-y (PMC10625973; doi:10.1245/s10434-023-14032-y)

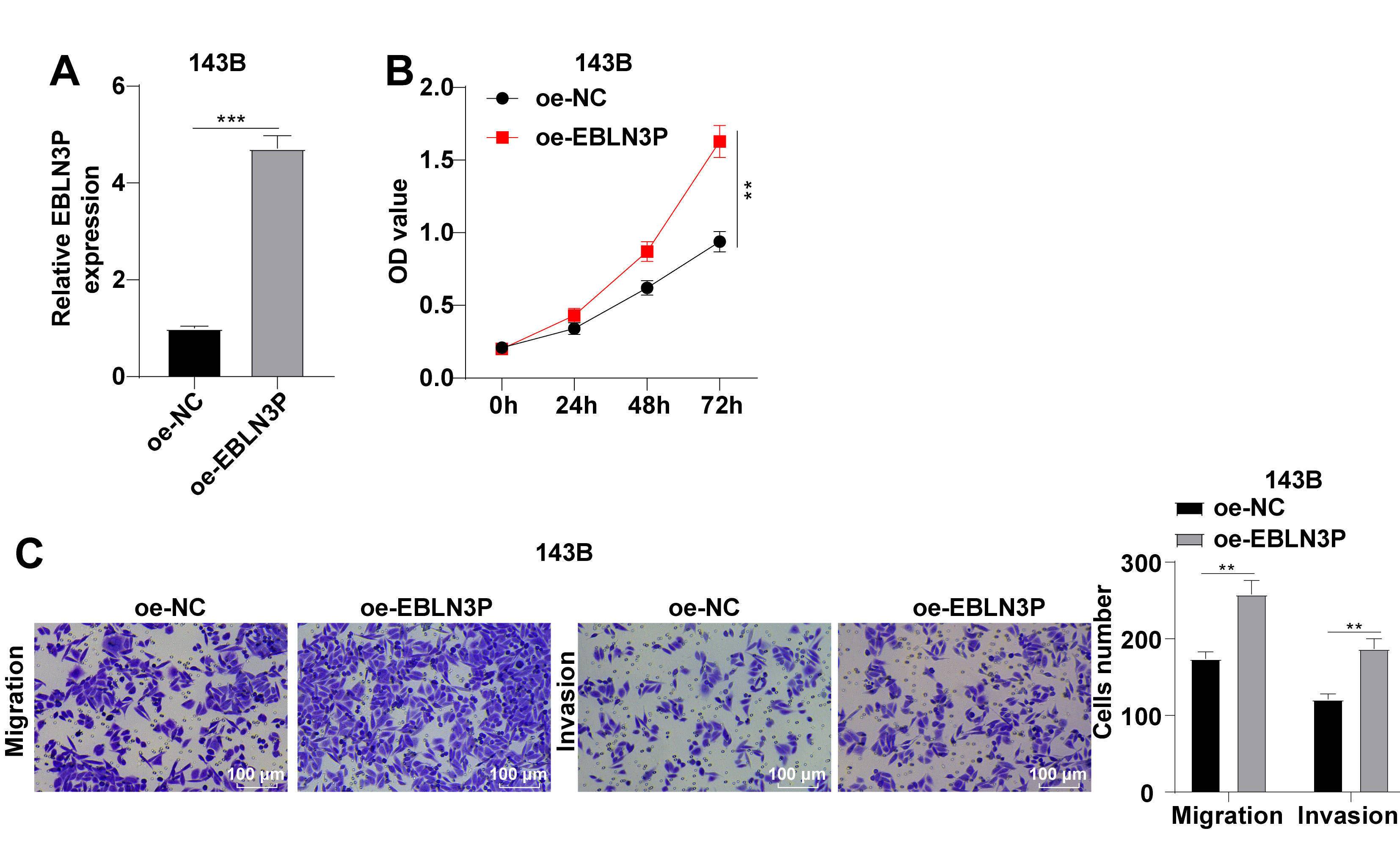

Supplement: Supplementary file 2 — Supplementary file2 (TIFF 6376 KB) [file 10434_2023_14032_MOESM2_ESM.tiff]
